# Low levels of serum urate are associated with a higher prevalence of depression in older adults: a nationwide cross-sectional study in Korea

**DOI:** 10.1186/s13075-020-02192-1

**Published:** 2020-05-06

**Authors:** Woo-Joong Kim, Hye Ri Kim, Jung Soo Song, Sang Tae Choi

**Affiliations:** 1 Division of Rheumatology, Department of Internal Medicine, Chung-Ang University College of Medicine, 102, Heukseok-ro, Dongjak-gu, Seoul, 06973 Republic of Korea; 2 Department of Psychiatry, Chung-Ang University College of Medicine, Seoul, Republic of Korea

**Keywords:** Depression, Korea National Health and Nutrition Examination Survey (KNHANES), Patient Health Questionnaire (PHQ)-9, Urate

## Abstract

**Background:**

Soluble urate has been shown to serve as an antioxidant, especially in the central nervous system. Although there are intriguing data suggesting that low levels of serum urate are associated with worse outcomes in neurodegenerative diseases, its impact on mental health has not been adequately assessed. Thus, we aimed to investigate the association between serum urate and depression using a large, nationally representative sample.

**Methods:**

Information on participants’ socio-demographic characteristics as well as physical and mental health conditions were retrieved from the Korea National Health and Nutrition Examination Survey (KNHANES) 2016 dataset. The Patient Health Questionnaire (PHQ)-9 was applied to identify depressive symptoms. Analyses were stratified by age: young adults (aged 19–39 years), middle-aged adults (aged 40–59 years), and older adults (aged 60 years and older).

**Results:**

A total of 5332 participants were included. Serum urate concentrations were divided into sex-specific quartiles based on their distribution: ≤ 4.9 (Q1), 5.0–5.7 (Q2), 5.8–6.6 (Q3), and ≥ 6.7 (Q4) mg/dL in men and ≤ 3.7 (Q1), 3.8–4.3 (Q2), 4.4–4.9 (Q3), and ≥ 5.0 (Q4) mg/dL in women. There was a significant negative linear relationship between serum urate quartiles and PHQ-9 scores in older adults (*p* for trend = 0.020 in men and *p* for trend = 0.048 in women). Compared to high levels (Q3 and Q4) of serum urate, low levels (Q1 and Q2) were significantly associated with the overall burden of depression in older women (OR 1.78, 95% CI 1.21, 2.61) and clinically relevant depression in older men (OR 3.35, 95% CI 1.16, 9.70), even after adjustment.

**Conclusions:**

Based on the KNHANES data, low levels of serum urate are associated with a higher prevalence of depression in older adults. This may have clinical implications for mental health.

## Background

Urate is a breakdown product of human purine metabolism which, at elevated serum concentrations, can cause gout, kidney stones, and acute kidney injury. The association of hyperuricemia with adverse health outcomes—such as metabolic syndrome, cardiovascular disease, and chronic kidney disease—has been described [1]. In general, a target serum urate concentration of no higher than 6.0 mg/dL would obtain meaningful benefit from urate-lowering therapy, while a serum urate concentration of 5.0 mg/dL has been considered the target in patients with gout exhibiting severe clinical features (polyarticular distribution, with established structural joint damage or tophi) [2]. These therapeutic targets were endorsed by multiple rheumatology societies worldwide [3–7]; however, the potential unintended consequences of therapy remain a concern. The British Society of Rheumatology advocates the possibility of adverse effects that may be associated with a very low serum urate level and the European League Against Rheumatism recommends against lowering serum urate concentrations below 3.0 mg/dL in the long-term. In contrast, other guidelines do not suggest any specific action to avoid inappropriate levels of serum urate [8].

Urate reduces the oxo-heme oxidant formed by a peroxide reaction with hemoglobin and protects erythrocytes from peroxidative damage leading to lysis [9]. It has been suggested that increased urate generation represented an evolutionary advantage towards enhanced antioxidant defenses that may have conferred a survival benefit [10]. A randomized, double-blinded, placebo-controlled study demonstrated that systemic urate infusion in healthy volunteers increased serum urate concentrations by 35% from 3.8 mg/dL to 5.2 mg/dL and was associated with a 23–139% increase in serum antioxidant capacity, depending on the assay used [11]. These effects are reported to be important in the central nervous system, in which acute administration of urate reduces neurological injury after ischemic stroke [12, 13]. Moreover, evidence suggested that higher levels of serum urate are associated with favorable neurological outcomes in Parkinson’s disease, Huntington’s disease, amyotrophic lateral sclerosis, and dementia [14–17]. However, there have been conflicting reports with the risk of stroke, dementia, especially vascular or mixed dementia, and Parkinson’s disease due to hyperuricemia and gout [18–20].

Over the last decade, it has been hypothesized that oxidative stress pathways might also be involved in the pathophysiological mechanism of depression [21]. It has been proposed that high levels of urate are associated with a lower risk of hospitalization due to depression and antidepressant medication use in two independent cohorts [22]. A recent meta-analysis and meta-regression provided additional evidence on this relationship, in which subjects with major depressive disorder had lower levels of serum urate than healthy controls [23]. However, no association between serum urate concentrations and behavioral and clinical characteristics was found in patients with major affective disorders [24]. As the role of serum urate is largely unclear in the development of depressive disorders, which have become a leading cause of health burden worldwide [25], this study aimed to investigate the association between serum urate concentrations and the prevalence of depression in a large, nationally representative sample drawn from the Korea National Health and Nutrition Examination Survey (KNHANES) 2016 dataset.

## Methods

### Study population

**Fig. 1:**
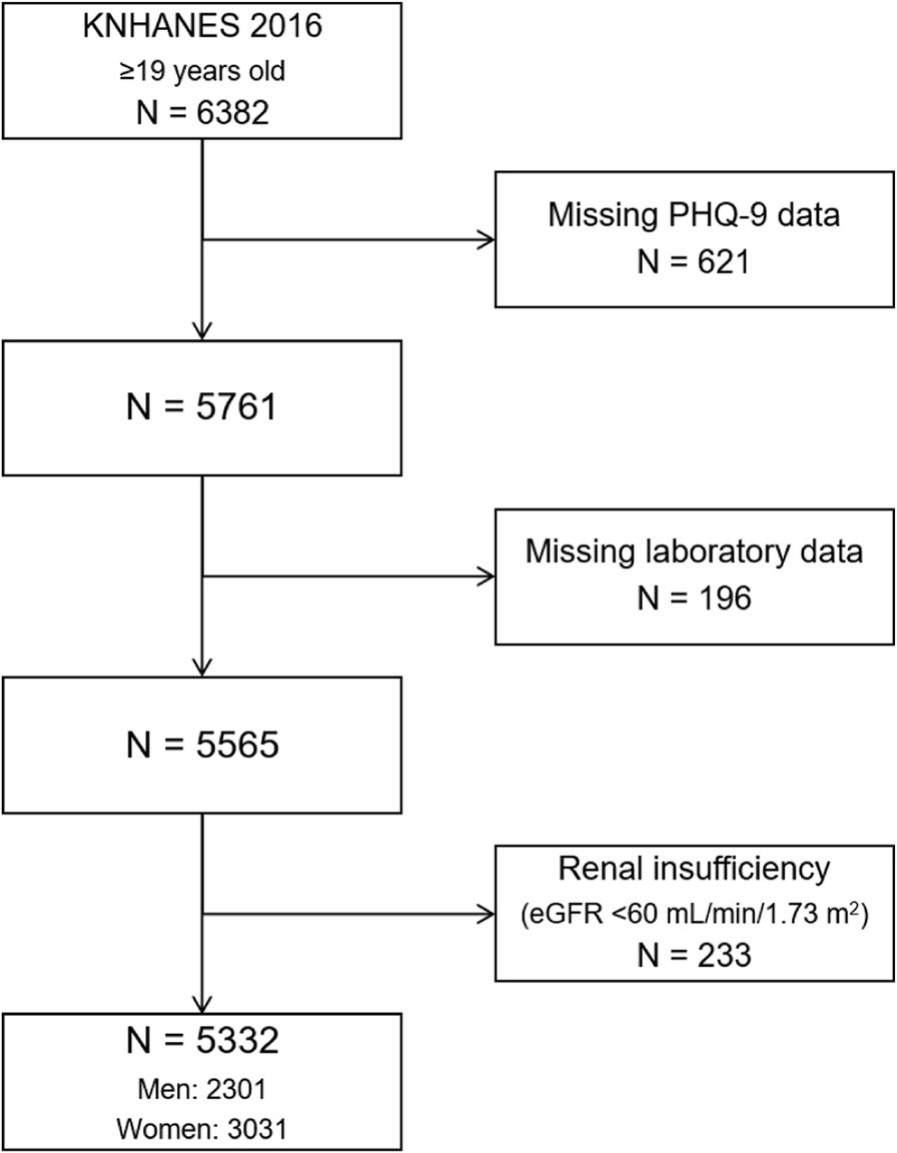
Study participants. eGFR, estimated glomerular filtration rate; KNHANES, Korea National Health and Nutrition Examination Survey; PHQ, Patient Health Questionnaire

The KNHANES is a cross-sectional, multistage, stratified, clustered probability sample survey of Korean civilians from a non-institutionalized population, conducted every year since 1998 by the Korea Centers for Disease Control and Prevention of the Ministry of Health and Welfare, designed to monitor the nation’s health and nutritional status. All consenting participants underwent a semi-structured interview and physical examination in a mobile examination center with blood and urine testing by standardized procedures, followed by a household interview. Each year, the survey involves a sample of roughly 7000–9000 individuals who are representative of Koreans. In the present study, we examined data from the publicly available KNHANES 2016 dataset of 8150 participants, in which serum urate concentrations were measured for the first time with the Patient Health Questionnaire (PHQ)-9 included as a screening instrument for depression. Exclusion criteria included age 18 years or younger (*n* = 1768), missing PHQ-9 data (*n* = 621), missing laboratory data (*n* = 196), and estimated glomerular filtration rate (eGFR) less than 60 mL/min/1.73 m^2^ (*n* = 233). The remaining 5332 subjects (2301 men and 3031 women) were eligible for the analysis (Fig. 1).

### Measurement of serum parameters

Blood samples were collected the morning after overnight fasting. In the chemistry panel, using a Hitachi automatic analyzer 7600-210 (Hitachi, Japan), serum urate and creatinine concentrations were measured by the calorimetric enzymatic method and the Jaffe rate-blanked and compensated method, respectively. Serum high-sensitivity C-reactive protein (CRP) concentration was measured by immunoturbidimetry using a Cobas automatic analyzer (Roche, Germany).

Since the glomerular filtration rate is usually accepted as the best overall index of kidney function, eGFR was calculated using the Chronic Kidney Disease Epidemiology Collaboration equation (eGFR = 141 × min (S_Cr_/*κ*, 1)^α^ × max (S_Cr_/*κ*, 1)^-1.209^ × 0.993^Age^ × 1.018 [if female] × 1.159 [if Black] mL/min/1.73 m^2^, where S_Cr_ is serum creatinine, *κ* is 0.7 for females and 0.9 for males, *α* is − 0.329 for females and − 0.411 for males, min indicates the minimum of S_Cr_/*κ* or 1, and max indicates the maximum of S_Cr_/*κ* or 1) [26].

### Assessment of demographic, socioeconomic, and health-related variables

This study collected information on the participants’ socio-demographic characteristics as well as physical and mental health conditions. The data were then stratified by age: young adults (aged 19–39 years), middle-aged adults (aged 40–59 years), and older adults (aged 60 years and older). Educational levels were categorized as elementary school, middle school, high school, or college, according to educational stages in Korea. Household income levels were calculated by dividing the total household monthly income by the square root of the household size; the obtained levels were then grouped into quartiles. Body mass index (BMI) was calculated as each participant’s weight in kilograms divided by the square of the height in meters. We classified subjects as underweight (BMI < 18.5 kg/m^2^), normal (BMI 18.5–24.9 kg/m^2^), and obese (BMI ≥ 25.0 kg/m^2^) according to the survey standard considering that the appropriate BMI cut-off for obesity in Asia is 25.0 kg/m^2^ [27]. The presence of chronic disease such as hypertension, diabetes, stroke, ischemic heart disease, thyroid disease, arthritis, pulmonary tuberculosis, asthma, chronic kidney disease, liver cirrhosis, and cancer was dichotomized into yes (whether the subject had been diagnosed by a physician) or no. Lifetime diagnosis of depression (yes/no), perceived health status (very good or good/fair, poor, or very poor), and perceived stress (no or mild/much or very much) were also dichotomized. Smoking status was categorized as current smoker (an adult who has smoked 100 cigarettes in his or her lifetime and who currently smokes cigarettes) or non-smoker (an adult who has never smoked, who has smoked less than 100 cigarettes in his or her lifetime, or who had quit smoking by the time of interview). For alcohol use, drinking status was categorized as current drinker (an adult who had consumed any kind of alcoholic beverage in the past year) or non-drinker.

### Patient Health Questionnaire (PHQ)-9

The PHQ-9 is a nine-item questionnaire designed to screen for depression, upon which the diagnosis of depressive disorders, as stated by the Diagnostic and Statistical Manual of Mental Disorders, Fourth Edition, is based. Participants were asked to indicate how often each depressive symptom had occurred during the preceding 2 weeks by choosing one of the following options: 0 (not at all), 1 (several days), 2 (more than half of the days), and 3 (nearly every day) [28]. The PHQ-9 has been validated to recognize not only major depression but also subthreshold depressive disorders in the general population and has been translated into several languages [29–32]. The Korean version of PHQ-9 was administered in the KNHANES with authorized permission. While a standard cut-off score of 10 has been proposed to maximize the combined sensitivity and specificity in a recent meta-analysis [33], a population-based prospective cohort study, which validated the Korean version of PHQ-9 in subjects older than 60 years, suggested that a score of 5 is the optimal cut-off for screening [34]. In this study, participants with a PHQ-9 score ≥ 5 or self-reported current depression were included to estimate the overall burden of depressive symptoms and those with a PHQ-9 score ≥ 10 were considered to have clinically relevant depression.

### Statistical analyses

All analyses of this 1-year sample were weighted and accounted for the stratified, multistage probability sampling design of the KNHANES 2016 and survey non-responses. Missing data were treated as valid among the strata, cluster, subpopulation, and factor variables and considered while estimating the prevalence. Descriptive statistics are presented using means or proportions with standard errors (SEs). Analysis of variance for continuous variables or the chi-square test for categorical variables was used to analyze the differences. The relationship between serum urate quartiles and the PHQ-9 score, as a continuous variable, was determined using the complex sample general linear models. The complex sample crosstabs procedure produced the weighted prevalence estimates for age groups to identify patterns in data. Then, the associations of serum urate quartiles with the prevalence of overall depression or clinically relevant depression, as categorical variables, were examined by the complex sample logistic regression models. Odds ratios (ORs) with 95% confidence intervals (CIs) were calculated from the corresponding models. All statistical tests were two-tailed, and *p* < 0.05 was considered significant. Data analyses were conducted using IBM SPSS Statistics for Windows, Version 23.0 (IBM Corp., Armonk, NY, USA).

## Results

### General characteristics

Among the 5332 subjects aged 19–80 years enrolled in this study, the mean serum urate concentration was 5.8 (SE = 0.03) mg/dL in 2301 men, which was significantly higher than the concentration of 4.3 (SE = 0.02) mg/dL in 3031 women (*p* < 0.001). Serum urate concentrations below 3.0 mg/dL were measured in 22 (0.7%) men and 181 (6.1%) women, while only 3 (0.1%) men and 10 (0.4%) women had concentrations less than 2.0 mg/dL. Serum urate concentrations were then divided into sex-specific quartiles based on their distribution: ≤ 4.9 (Q1), 5.0–5.7 (Q2), 5.8–6.6 (Q3), and ≥ 6.7 (Q4) mg/dL in men and ≤ 3.7 (Q1), 3.8–4.3 (Q2), 4.4–4.9 (Q3), and ≥ 5.0 (Q4) mg/dL in women.

Table 1 describes the general characteristics of the study population according to serum urate quartiles. A decrease in the mean age was associated with an increase in serum urate concentrations in men. An increasing trend in BMI was observed across the quartiles in both the sexes. In men, the proportions of individuals with a lifetime diagnosis of depression, self-reported current depression, and current antidepressant use were higher in the fourth quartile (Q4). The prevalence of depression (PHQ-9 score ≥ 5 or self-reported current depression) was substantially higher among women than among men (25.2% vs. 15.5%, *p* < 0.001). It was also noted that clinically relevant depression (PHQ-9 score ≥ 10) was significantly more prevalent among women than among men (7.0% vs. 3.9%, *p* < 0.001). In the entire sample, there were no significant relationships between serum urate quartiles and PHQ-9 scores.

**Table 1:** General characteristics of participants

Variables	Men (***n*** = 2301)						Women (***n*** = 3031)					
	1st quartile	2nd quartile	3rd quartile	4th quartile	Total	***p***	1st quartile	2nd quartile	3rd quartile	4th quartile	Total	***p***
Age, years												
Mean	50.0 (0.7)	45.3 (0.8)	43.3 (0.8)	41.5 (0.6)	45.0 (0.4)	< 0.001	47.2 (0.7)	45.8 (0.7)	46.4 (0.8)	47.3 (0.8)	46.6 (0.5)	0.378
Categories						< 0.001						0.059
Young adults (19–39)	25.5 (2.3)	39.5 (2.8)	44.0 (2.5)	47.5 (2.4)	39.2 (1.4)		31.5 (2.1)	37.7 (2.1)	37.6 (2.3)	37.1 (2.4)	35.8 (1.3)	
Middle-aged adults (40–59)	48.0 (2.5)	41.4 (2.3)	39.0 (2.2)	40.3 (2.4)	42.1 (1.3)		45.9 (2.0)	41.5 (2.1)	41.6 (1.9)	37.9 (2.3)	41.9 (1.1)	
Older adults (60+)	26.5 (2.0)	19.1 (1.6)	16.9 (1.5)	12.3 (1.2)	18.7 (0.9)		22.5 (1.6)	20.9 (1.6)	20.9 (1.7)	25.0 (1.8)	22.3 (1.0)	
Educational levels						< 0.001						0.652
Elementary school	14.1 (1.5)	9.7 (1.2)	7.3 (1.0)	5.1 (0.8)	9.0 (0.6)		17.8 (1.6)	19.5 (1.7)	17.7 (1.7)	19.0 (1.8)	18.5 (1.0)	
Middle school	10.5 (1.3)	6.9 (1.1)	6.9 (1.2)	8.8 (1.4)	8.3 (0.7)		10.5 (1.3)	8.6 (1.1)	9.7 (1.2)	9.7 (1.4)	9.6 (0.6)	
High school	36.2 (2.4)	36.7 (2.5)	36.4 (2.2)	40.0 (2.6)	37.3 (1.4)		38.8 (1.9)	33.9 (1.9)	36.6 (2.3)	34.3 (2.3)	36.0 (1.1)	
College	39.1 (2.6)	46.7 (2.7)	49.4 (2.5)	46.1 (2.6)	45.4 (1.7)		32.9 (2.1)	38.0 (2.1)	35.9 (2.8)	37.0 (2.3)	35.9 (1.3)	
Household income levels						0.381						0.817
1st quartile	15.5 (1.9)	14.3 (2.0)	12.8 (1.6)	12.2 (2.0)	13.7 (1.2)		15.4 (1.6)	15.0 (1.6)	17.0 (1.8)	17.9 (2.2)	16.2 (1.2)	
2nd quartile	20.6 (2.2)	24.9 (2.2)	20.9 (2.0)	23.9 (2.3)	22.6 (1.2)		24.0 (1.9)	22.0 (2.0)	23.5 (1.9)	24.3 (1.9)	23.4 (1.1)	
3rd quartile	33.8 (2.3)	29.1 (2.3)	32.5 (2.5)	28.0 (2.5)	30.8 (1.4)		30.7 (2.1)	32.3 (2.1)	28.7 (2.1)	27.5 (2.2)	29.9 (1.3)	
4th quartile	30.1 (2.7)	31.7 (2.8)	33.8 (2.6)	36.0 (2.7)	32.9 (1.8)		29.9 (2.4)	30.7 (2.6)	30.9 (2.6)	30.3 (2.5)	30.5 (1.8)	
Body mass index, kg/m^2^												
Mean	24.0 (0.2)	23.9 (0.1)	24.7 (0.2)	25.8 (0.2)	24.6 (0.1)	< 0.001	22.6 (0.1)	22.9 (0.1)	23.4 (0.2)	24.7 (0.2)	23.4 (0.1)	< 0.001
Categories						< 0.001						< 0.001
< 18.5	3.4 (0.8)	3.3 (1.1)	1.9 (0.8)	1.2 (0.5)	2.5 (0.4)		6.3 (1.0)	5.6 (0.9)	6.3 (1.3)	4.0 (0.9)	5.6 (0.6)	
18.5–24.9	61.5 (2.3)	61.7 (2.2)	54.3 (2.3)	44.4 (2.5)	55.5 (1.3)		74.1 (1.7)	71.9 (1.8)	65.5 (2.0)	51.2 (2.1)	66.1 (1.0)	
≥ 25	35.1 (2.4)	34.9 (2.3)	43.8 (2.3)	54.4 (2.5)	42.1 (1.3)		19.6 (1.6)	22.5 (1.6)	28.3 (1.8)	44.8 (2.2)	28.3 (1.1)	
Presence of chronic disease	40.6 (2.4)	32.6 (2.0)	27.9 (2.0)	26.8 (2.2)	31.9 (1.1)	< 0.001	34.1 (1.9)	32.6 (1.9)	35.4 (2.2)	42.5 (2.3)	36.0 (1.2)	0.004
Lifetime diagnosis of depression	2.2 (0.7)	2.8 (0.8)	0.7 (0.3)	4.2 (1.1)	2.5 (0.4)	0.007	5.8 (1.1)	6.5 (0.9)	5.2 (1.0)	5.2 (1.0)	7.3 (1.2)	0.566
Perceived health status						0.877						0.030
Fair, poor, or very poor	65.1 (2.4)	63.3 (2.2)	64.8 (2.4)	65.9 (2.2)	64.8 (1.1)		74.8 (1.8)	68.5 (1.9)	68.2 (2.1)	72.8 (2.0)	71.1 (1.1)	
Perceived stress						0.127						0.513
Much or very much	25.0 (2.0)	26.0 (2.1)	26.2 (2.0)	31.9 (2.4)	27.3 (0.9)		29.5 (1.9)	27.5 (1.8)	27.6 (2.1)	31.2 (2.1)	28.9 (0.9)	
Current smokers	40.0 (2.3)	36.8 (2.6)	41.0 (2.7)	40.0 (2.4)	39.5 (1.4)	0.637	3.9 (0.7)	5.6 (1.0)	6.8 (1.2)	9.0 (1.5)	6.2 (0.6)	0.008
Current drinkers	84.4 (1.5)	84.5 (1.8)	91.1 (1.3)	90.1 (1.4)	87.6 (0.8)	0.001	65.6 (1.8)	70.9 (1.9)	71.2 (2.0)	70.7 (1.9)	69.5 (1.1)	0.091
Self-reported current depression	1.0 (0.5)	1.0 (0.4)	0.5 (0.3)	2.7 (0.9)	1.3 (0.3)	0.013	3.4 (0.8)	4.1 (0.8)	2.8 (0.7)	4.6 (1.0)	3.7 (0.4)	0.794
Current antidepressant use	1.0 (0.5)	0.4 (0.2)	0.3 (0.3)	2.3 (0.9)	1.0 (0.3)	0.004	2.4 (0.7)	2.8 (0.7)	1.7 (0.5)	3.2 (0.7)	2.5 (0.3)	0.807
PHQ-9 scores												
Mean	2.1 (0.2)	2.0 (0.2)	1.9 (0.2)	2.3 (0.2)	2.1 (0.1)	0.660	3.1 (0.2)	3.2 (0.2)	3.1 (0.2)	3.3 (0.2)	3.2 (0.1)	0.748
≥ 5	15.8 (1.9)	14.5 (1.9)	12.9 (1.7)	17.1 (2.0)	15.0 (0.9)	0.399	23.7 (1.7)	25.1 (1.8)	23.8 (2.1)	26.0 (1.9)	24.7 (1.1)	0.762
≥ 10	3.7 (0.8)	4.0 (1.0)	3.5 (0.8)	4.3 (1.2)	3.9 (0.5)	0.927	6.9 (1.0)	7.1 (1.1)	6.5 (1.1)	7.5 (1.3)	7.0 (0.6)	0.932
CRP, mg/L, mean	1.52 (0.14)	1.14 (0.10)	1.17 (0.08)	1.50 (0.10)	1.33 (0.05)	0.007	0.93 (0.07)	0.86 (0.05)	1.10 (0.08)	1.43 (0.09)	1.08 (0.04)	< 0.001
eGFR, mL/min/1.73 m^2^, mean	96.5 (0.8)	96.6 (0.9)	97.0 (0.8)	95.8 (0.9)	96.5 (0.5)	0.759	103.8 (0.7)	102.5 (0.6)	100.2 (0.7)	97.7 (0.8)	101.1 (0.4)	< 0.001

Values are presented using means or proportions with standard errors

*BMI* body mass index, *CRP* C-reactive protein, *eGFR* estimated glomerular filtration rate, *EQ-5D* EuroQol-5 Dimensions, *PHQ* Patient Health Questionnaire

### Age-stratified relationships between serum urate quartiles and PHQ-9 scores

General linear models with polynomial contrasts were used to test whether a linear trend is present between serum urate quartiles and adjusted PHQ-9 scores. Table 2 shows age-stratified relationships across the serum urate quartiles, after controlling for several socio-demographic, health-, and disease-related variables, such as age, educational levels, household income levels, categorized BMI, the presence of chronic disease, lifetime diagnosis of depression, perceived health status, perceived stress, smoking status, and drinking status. These models revealed a significant negative linear effect for adjusted PHQ-9 scores in older men (*p* for linear trend = 0.020) and older women (*p* for linear trend = 0.048). The results indicated a higher PHQ-9 score among older adults with low levels of serum urate, but this association was not valid for young or middle-aged adults.

**Table 2:** Age-stratified relationships between serum urate quartiles and PHQ-9 scores

	**Young men (19–39 years,*n*** **= 712)**				**Middle-aged men (40–59 years,*n*** **= 879)**				**Older men (60 years and older,*n*** **= 710)**				**Men (*n*** **= 2301)**			
	**Mean**	**SE**	***F***	***p***	**Mean**	**SE**	***F***	***p***	**Mean**	**SE**	***F***	***p***	**Mean**	**SE**	***F***	***p***
**1st quartile**	6.47	1.33	0.029	0.865	3.76	0.44	0.071	0.790	4.02	0.54	5.448	0.020	4.44	0.46	0.169	0.681
**2nd quartile**	6.12	1.28			4.00	0.48			3.75	0.54			4.26	0.44		
**3rd quartile**	6.37	1.28			3.68	0.45			3.65	0.52			4.27	0.43		
**4th quartile**	6.45	1.32			3.78	0.52			3.23	0.53			4.35	0.46		
	**Young women (19–39 years,*n*** **= 938)**				**Middle-aged women (40–59 years,*n*** **= 1184)**				**Older women (60 years and older,*n*** **= 909)**				**Women (*n*** **= 3031)**			
	**Mean**	**SE**	***F***	***p***	**Mean**	**SE**	***F***	***p***	**Mean**	**SE**	***F***	***p***	**Mean**	**SE**	***F***	***p***
**1st quartile**	5.75	0.85	0.867	0.352	6.24	0.58	0.087	0.764	7.14	0.89	3.928	0.048	6.48	0.41	0.180	0.894
**2nd quartile**	6.43	0.86			6.21	0.56			6.78	0.86			6.68	0.39		
**3rd quartile**	6.55	0.90			5.93	0.57			6.69	0.86			6.60	0.40		
**4th quartile**	6.03	0.83			6.25	0.67			6.29	0.82			6.48	0.42		

*F* and *p* values are calculated for linear trend

*PHQ* Patient Health Questionnaire, *SE* standard error

### Age-stratified prevalence of depression

**Fig. 2:**
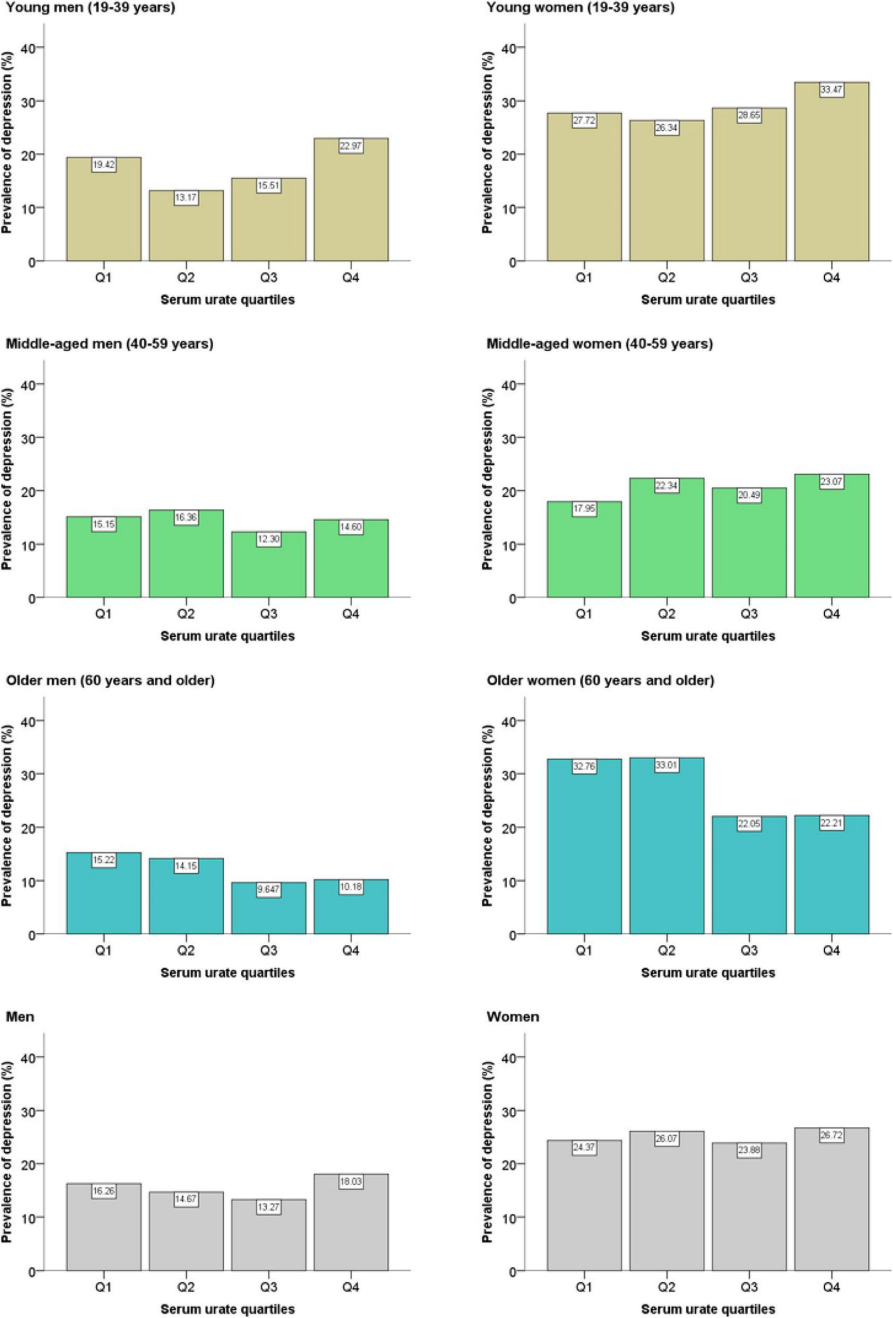
Prevalence of overall depression according to serum urate quartiles

Figure 2 depicts various patterns of weighted prevalence estimates for the overall depression according to serum urate quartiles. These data indicated a U-shaped trend for depression among young adults. No significant trend was detected among middle-aged adults. While the PHQ-9 scores themselves showed a linear trend that decreases with serum urate quartiles, when comparing the overall burden of depressive symptoms in older adults, a given population can be roughly divided into two equal halves. It has been demonstrated that individuals with low levels of serum urate (Q1 and Q2 rather than Q3 and Q4) have a relatively high prevalence of depression, although the difference is more pronounced in women. It was noted that the fourth quartile (Q4) had a higher proportion of subjects with depression than the third quartile (Q3) in every subgroup.

**Fig. 3:**
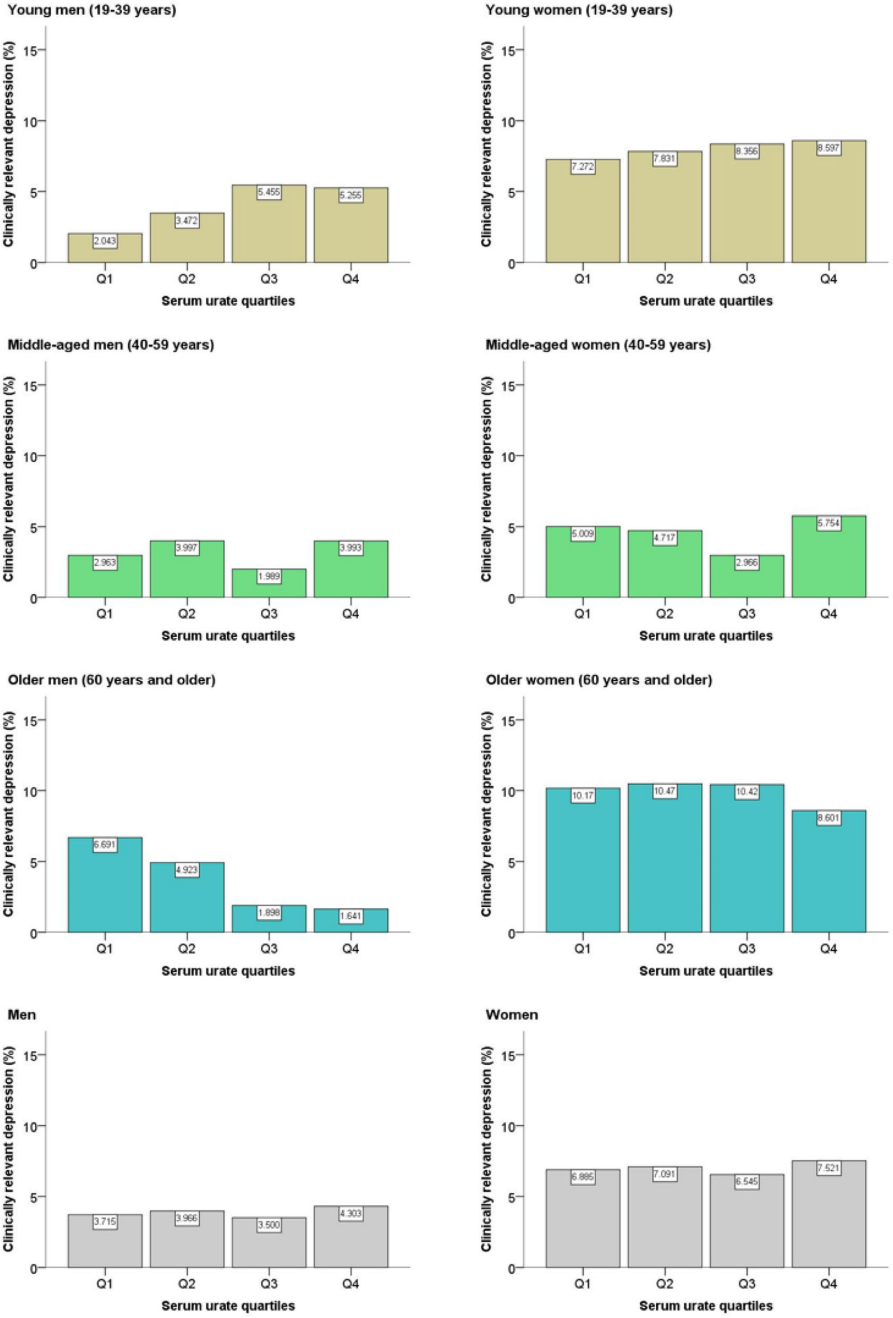
Prevalence of clinically relevant depression according to serum urate quartiles

Figure 3 shows a plot of weighted prevalence estimates for clinically relevant depression, which represents a more specific and serious condition, according to serum urate quartiles. In contrast to the U-shaped trend of the overall depression prevalence, an upward trend was observed among young adults of both sexes. In men, the prevalence of clinically relevant depression differed according to low levels (Q1 and Q2) and high levels (Q3 and Q4) of serum urate.

### Association between serum urate levels and the prevalence of depression

Logistic regression methods were used to examine whether these differences in the prevalence of overall depression according to low and high levels of serum urate were independent of confounding variables. If the association is indeed present, the models estimated adjusted ORs in older men and women. Additionally, these analyses were repeated for clinically relevant depression in older adults.

Table 3 presents the independent associations of factors with the prevalence of overall depression. Socio-demographic, health-, and disease-related variables were assigned to build a statistical model. In accordance with the crude analysis, low levels (Q1 and Q2), compared to high levels (Q3 and Q4), of serum urate were identified as a significant factor for the prevalence of overall depression (OR 1.78, 95% CI 1.21, 2.61) in older women, along with the presence of chronic disease, lifetime diagnosis of depression, poor perceived health status, and perceived stress.

**Table 3:** Association of serum urate levels with overall depression in older adults

	Older men (*n* = 710)		Older women (*n* = 909)	
	Odds ratio	95% CI	Odds ratio	95% CI
**Crude analysis**				
Urate levels				
Low (Q1, Q2)	1.58	0.83, 3.04	**1.72**	**1.26, 2.36**
High (Q3, Q4)	Reference		Reference	
**Adjusted analysis**				
Age (per 1 year)	1.01	0.97, 1.06	1.00	0.97, 1.04
CRP (per 1 mg/L)	1.02	0.96, 1.08	1.00	0.95, 1.05
Urate levels				
Low (Q1, Q2)	1.44	0.74, 2.80	**1.78**	**1.21, 2.61**
High (Q3, Q4)	Reference		Reference	
Educational levels				
Elementary school	1.65	0.66, 4.14	0.90	0.41, 2.00
Middle school	1.22	0.39, 3.81	1.36	0.59, 3.14
High school	0.65	0.21, 1.98	1.65	0.71, 3.81
College	Reference		Reference	
Household income levels				
1st quartile	**5.82**	**1.56, 21.77**	1.42	0.76, 2.65
2nd quartile	**4.34**	**1.00, 18.73**	0.89	0.49, 1.62
3rd quartile	1.94	0.48, 7.92	0.90	0.47, 1.74
4th quartile	Reference		Reference	
Categorized BMI				
< 18.5	1.87	0.84, 4.17	2.21	0.71, 6.88
18.5–24.9	Reference		Reference	
≥ 25	0.66	0.35, 1.26	0.87	0.56, 1.36
Presence of chronic disease				
No	Reference		Reference	
Yes	1.15	0.56, 2.34	**1.67**	**1.04, 2.70**
Lifetime diagnosis of depression				
No	Reference		Reference	
Yes	**62.37**	**8.43, 461.35**	**13.84**	**6.62, 28.91**
Perceived health status				
Very good or good	Reference		Reference	
Fair, poor, or very poor	**4.81**	**2.39, 9.69**	**2.91**	**1.49, 5.69**
Perceived stress				
No or mild	Reference		Reference	
Much or very much	**5.46**	**2.84, 10.50**	**3.79**	**2.48, 5.79**
Smoking status				
Non-smoker	Reference		Reference	
Current smoker	1.14	0.61, 2.12	1.27	0.47, 3.44
Drinking status				
Non-drinker	Reference		Reference	
Current drinker	1.15	0.62, 2.12	0.91	0.60, 1.39

All values are rounded to two decimal places

Bold values denote statistical significance

*BMI* body mass index, *CI* confidence interval, *CRP* C-reactive protein

The results shown in Table 4 are from the model for the prevalence of clinically relevant depression. Compared to that of high levels (Q3 and Q4), the association of low levels (Q1 and Q2) of serum urate retained significance (OR 3.35, 95% CI 1.16, 9.70) following adjustment in men.

**Table 4:** Association of serum urate levels with clinically relevant depression in older adults

	Older men (*n* = 710)		Older women (*n* = 909)	
	Odds ratio	95% CI	Odds ratio	95% CI
**Crude analysis**				
Urate levels				
Low (Q1, Q2)	**3.47**	**1.34, 8.96**	1.10	0.69, 1.76
High (Q3, Q4)	Reference		Reference	
**Adjusted analysis**				
Age (per 1 year)	1.01	0.93, 1.08	1.01	0.96, 1.07
CRP (per 1 mg/L)	1.03	0.94, 1.12	0.98	0.90, 1.06
Urate levels				
Low (Q1, Q2)	**3.35**	**1.16, 9.70**	1.15	0.67, 1.98
High (Q3, Q4)	Reference		Reference	
Educational levels				
Elementary school	0.96	0.19, 4.79	2.41	0.32, 17.99
Middle school	0.64	0.12, 3.32	2.66	0.29, 24.74
High school	0.32	0.05, 2.21	3.12	0.32, 30.56
College	Reference		Reference	
Household income levels				
1st quartile	–^a^		–^a^	
2nd quartile	–^a^		–^a^	
3rd quartile	–^a^		–^a^	
4th quartile	Reference		Reference	
Categorized BMI				
< 18.5	0.83	0.20, 1.25	0.87	0.13, 5.84
18.5–24.9	Reference		Reference	
≥ 25	0.45	0.16, 1.25	1.11	0.66, 1.87
Presence of chronic disease				
No	Reference		Reference	
Yes	3.34	0.87, 12.79	**2.92**	**1.31, 6.53**
Lifetime diagnosis of depression				
No	Reference		Reference	
Yes	1.15	0.23, 5.64	**5.14**	**2.58, 10.26**
Perceived health status				
Very good or good	Reference		Reference	
Fair, poor, or very poor	**13.42**	**1.60, 112.24**	**11.90**	**2.12, 66.84**
Perceived stress				
No or mild	Reference		Reference	
Much or very much	**8.67**	**3.72, 20.21**	**5.91**	**3.26, 10.73**
Smoking status				
Non-smoker	Reference		Reference	
Current smoker	1.17	0.45, 3.06	**3.91**	**1.23, 12.49**
Drinking status				
Non-drinker	Reference		Reference	
Current drinker	1.69	0.64, 4.46	**0.57**	**0.33, 0.99**

All values are rounded to two decimal places

Bold values denote statistical significance

*BMI* body mass index, *CI* confidence interval, *CRP* C-reactive protein

^a^Values are 0 or indefinite, as there were no or a small number of exposed cases and/or control subjects

## Discussion

To the best of our knowledge, this study is the first to highlight the association between the levels of serum urate and depression using a large, national-level database, which provided unbiased and comprehensive data of the Korean population. We demonstrated that low levels of serum urate are significantly associated with a higher PHQ-9 score and a higher prevalence of depression in older adults, even when the concentrations were within the physiological range.

Depression in older adults is associated with disability, increased mortality, and poorer outcomes of physical illness [35]; it is often inadequately treated and associated with higher relapse rates [36]. Though it is not fully understood, several etiological factors in late-life depression, especially related to aging and disease-related processes, have been shown to begin during mid-life [37], and patients with depression displayed a significantly enhanced senescent-associated secretory phenotype compared with controls [38]. Therefore, it is imperative to understand the life course perspectives on the epidemiology of depression and adhere to the United States Preventive Services Task Force’s recommendation for screening for depression while calling for more research on optimal screening approaches in older adults [39].

Age-stratified analyses in our study could allow the distinction between different patterns of depression among young, middle-aged, and older adults. Given the results, the possible neuroprotective effect of urate against depressive disorders, whereby urate acts as a potent antioxidant in the extracellular environment and accounts for nearly two thirds of total serum antioxidant activity [40], may be modulated by aging, although the potential biological mechanisms underlying the observed associations are speculative. A possible rationale might be found in the meta-analysis of 48 studies involving 9203 individuals that showed an association between microvascular dysfunction and late-life depression; this can be explained, at least in part, by a high level of reactive oxygen species produced in the brain as a result of the higher metabolic demand [41]. Another study, in which plasma levels of six oxidative stress markers were measured, provided robust evidence that subjects with late-life depression exhibit a significant imbalance in the oxidative stress response [42].

Inverse associations between serum urate levels and the overall burden of depressive symptoms were significant in older women but not in older men. Although apparent differences in the prevalence of depression according to low and high levels of serum urate were shown in both sexes, a possible explanation might be that the absolute difference in our study was much smaller for men. In addition, the statistical results might be less significant because of the lower prevalence of depression in men than in women. Nonetheless, serum urate levels may still considerably affect older men, as low levels of serum urate triple the odds of clinically relevant depression. Therefore, it seems logical that the association between low levels of serum urate and the overall burden of depressive symptoms in our study may underrepresent that in older men.

In line with the prior observations of a U-shaped curve of serum urate concentrations and mortality [43–46], a similar pattern was found for the overall depression prevalence among young adults. In contrast, those with high levels of serum urate were more likely to have clinically relevant depression; however, this was not significant after adjustment for socio-demographic, health-, and disease-related variables. More evidence will be required to determine whether depression in young adults is associated with serum urate concentrations. Further, the observation of a higher prevalence of depression in the fourth quartile than in the third quartile may have resulted from the previous finding that patients with gout are likely to experience depressive symptoms [47, 48].

Concerns about the possible detrimental effects of urate-lowering therapy have been gradually raised in line with recent increases in demands for the treatment of asymptomatic hyperuricemia and cardiovascular and/or kidney diseases [49, 50], as well as gout. Since novel uricosurics, in addition to potent inhibitors of xanthine oxidase, are expected to provide an efficient and reliable means of urate-lowering [51], there is a need for better understanding of the adverse effects due to low levels of serum urate. However, it is worth acknowledging that the implications of this epidemiological study need to be carefully interpreted with regard to low serum urate levels induced by pharmacological interventions, because it can be assumed that most participants from the general population were not receiving urate-lowering therapy in this study. Future high-quality research on the effects of urate-lowering therapy on neurological and mental health will help clarify such issues.

It is also notable that 25% of male participants and 75% of female participants in this study population had serum urate concentrations below 5 mg/dL, which has been conveniently used as an initial therapeutic target for the management of gout, but few patients had concentrations below 3 mg/dL. This means that maintaining relatively low levels in the physiological range but not necessarily very low levels of serum urate may be accompanied by undesirable effects. While this study was conducted in the general population and the importance of the effective management of gout cannot be overstated, our findings may support the use of dose reduction of urate-lowering therapy to less stringent targets after stabilization, as advocated by the British Society for Rheumatology and the European League Against Rheumatism [5, 6].

As urate is excreted primarily by the kidneys, elevated serum urate concentrations are strongly correlated with impaired renal function (eGFR < 60 mL/min/1.73 m^2^) [52]. Based on previous research using the KNHANES dataset and provided that the prevalence of depression increased with chronic kidney disease stage 3 or greater [53, 54], the exclusion of these subjects (*n* = 233) from this study could suggest that the association between serum urate levels and the prevalence of depression is unlikely to be related to renal function. However, this could potentially cause bias in the generalizability of the results when, for instance, a considerable number of subjects have impaired renal function.

Data from this cross-sectional study provide a single snapshot in time of serum urate levels and depression status and have a limited capacity to examine any causal relationship. Further clinical and experimental studies will be needed to demonstrate the temporal aspects of the relationship between serum urate levels and depression symptomatology and to investigate the potential mechanistic role of serum urate in the natural history of depressive disorders. Although participants taking an antidepressant for major depressive disorder or other indications may have exhibited few depressive symptoms, it was not possible to determine whether PHQ-9 scores are influenced by prior exposure to antidepressants. In addition, most symptom-screening questionnaires including the PHQ-9 were not designed to ascertain diagnostic status and would theoretically be expected to overestimate prevalence [55]. However, these tools have important applications for the assessment of symptom severity, regardless of the diagnostic status, and as screening tools to identify people who may have depression [56]. There is also an interest in the field of bipolar and related disorders, as shown in a meta-analysis reported that subjects with bipolar disorder had higher serum urate concentrations than healthy controls [57]. It is possible that a portion of patients with bipolar disorder experiencing depression was also included in the analysis.

## Conclusions

Our findings suggest that low levels of serum urate are associated with a higher prevalence of depression in older adults. Longitudinal studies are needed to examine the potential effects of serum urate on the trajectory of depression over time. This may have clinical implications for mental health.

## Data Availability

The dataset used and/or analyzed during the current study is available from the Korea National Health and Nutrition Examination Survey website (https://knhanes.cdc.go.kr/).

## Abbreviations

BMI: Body mass index
CI: Confidence interval
CRP: C-reactive protein
eGFR: Estimated glomerular filtration rate
KNHANES: Korea National Health and Nutrition Examination Survey
OR: Odds ratio
PHQ: Patient Health Questionnaire
SE: Standard error
